# Effectiveness and safety of light vegetarian diet on functional constipation with gastrointestinal damp-heat pattern: An exploratory study protocol for randomized controlled trial

**DOI:** 10.1097/MD.0000000000018325

**Published:** 2019-12-16

**Authors:** Yu Liu, Xudong Gao, Yuehua Ding, Yuanchen Zhou, Xinyuan Liu, Huijing Wang, Qianqian Wang, Bingzhi Ma, Shukun Yao

**Affiliations:** a School of Graduates, Beijing University of Chinese Medicine; b Department of Gastroenterology of Traditional Chinese Medicine, China-Japan Friendship Hospital; c Department of Endoscopy Center, Beijing Rectum Hospital; d Peking University China-Japan Friendship School of Clinical Medicine, Peking University; e Department of Pharmacy, China-Japan Friendship Hospital; f Department of Gastroenterology, China-Japan Friendship Hospital, Beijing, China.

**Keywords:** diet therapy, exploratory study, functional constipation, randomized controlled trial, traditional Chinese medicine

## Abstract

**Introduction:**

Functional constipation (FC) is one of the common gastrointestinal disorders that affects people of almost every age. Persistent FC significantly affects quality of life and well-being along with economic burden on patients as well as health care system. Therapeutic efficacy of currently used treatment strategies becomes limited shortly after their discontinuation as constipation occurs again as a result of inappropriate dietary habits. Previous studies have revealed that light vegetarian diet (LVD) can significantly improve both typical and atypical subtypes of major traditional Chinese medicine (TCM) FC syndrome such as gastrointestinal damp-heat syndrome. This protocol aims at exploratorily investigating effectiveness and safety of LVD following a rigorous clinical trial.

**Methods and design:**

Total 92 patients in each of the 2 subtypes will be recruited in China-Japan Friendship Hospital for participating in this prospective, placebo-controlled, randomized trial and exploratory study. The patients in each subtype will be randomly divided into 4 groups according to 1:1:1:1 ratio with allocation concealment, which are drug + diet group, drug group, placebo + diet group and placebo group. Patients in the group with diet intervention will be required to strictly follow the LVD. The study will continue for a period of 28 days, including a drug or placebo supervised intervention and a 14th-day telephone follow-up. During the intervention, patients will be required to record a designed diary for controlling the diet quality (DQ) and analyzing the defecation. The study will focus investigation of complete spontaneous bowel movements (CSBM) per week as its primary outcome and constipation-related symptom rating scale (CSS), TCM syndrome scale (TCMSS), 48-hour gastrointestinal transit time (48-hour GITT), high resolution anorectal manometry (HRAM) and fecal flora detection (FFD) will be included in secondary outcomes. Furthermore, the study will also determine safety, DQ and compliance indicators.

**Ethics and dissemination:**

This study has been approved by China-Japan Friendship Hospital clinical research ethics committee (No. 2017–46–1). A SPIRIT checklist is available for this protocol.

**Trial registration number:**

ChiCTR1800019686 in Chinese Clinical Trial Registry (WHO ICTRP member).

## Abstract 2

Supplemental Digital Content is available in the text

## 1 Introduction

FC is one of the common functional gastrointestinal disorders that affects a large population around the globe. Prevalence rate of FC varies greatly from 1.9% to 27.2% in North America population with the highest estimates from 12% to 19%;^[1]^ In China, it varies from 6% to 13% for the period of 2010 to 2016.^[2,3]^ Gender, age, diet, education, and neurological diseases are the main influencing factors for FC.^[4,5]^ FC is protracted and prone to recurrence, thus affects patient's quality of life adversely along with increase in overall medical costs.^[6]^

Prolonged colonic transit time (slow transit constipation, STC), pelvic floor muscles dysfunction (functional defecation disorder) and their combined effects are the well-established mechanisms involved in the development of FC. The latest diagnosis of FC is based on the criteria of Rome IV Standard, including laborious defecation, dry or lumpy stool, incomplete defecation, anorectal obstruction, prolonged defecation cycle, and manually assisted defecation. Two or more of the above items need to be included with at least 25% frequency for each one. Meanwhile, loose stool is rarely present without laxative and irritable bowel syndrome (IBS) should be excluded.^[7]^ Dry or lumpy stool is not necessary for FC diagnosis as many patients whose stool is not that dry or lumpy but sticky, complain of difficult defecation. However, there are few studies on this kind of constipation. In TCM, gastrointestinal damp-heat syndrome is the major FC syndrome that accounts for almost 32.5% FC cases.^[8]^ Heat evil results in formation of dry or lumpy stool, which is in agreement with typical understanding of FC in western medicine, while damp evil inclines to cause sticky stool in TCM theory. Based on that, the classification therapy shows greater therapeutic efficacy against FC with gastrointestinal damp-heat syndrome. Thus, we can divide this FC syndrome into 2 subtypes that are heat more than damp syndrome (typical functional constipation, TFC) and damp more than heat syndrome (atypical functional constipation, aTFC). Drugs therapy is still the main method for effective FC treatment. For TFC, Macrogol 4000 Powder is one of the effective and safe drugs, which can increase the average number of defecation per week by 1.9 times as compared to placebo.^[9]^ On other hand, the effectiveness of Chinese herb for aTFC could reach 66.7% to 72.7%.^[10]^ However, it has been recently reported that only 25.1% among 6318 Chinese adults with constipation selected laxatives, while 81.7% of them preferred lifestyle and dietary changes as treatment option.^[11]^ The results of a current study have shown a close relationship between the intestinal flora and FC,^[12]^ but their causal relationship still remains unclear.^[13]^ Different eating habits such as low intake of water, fruits and vegetables and high intake of spicy, greasy food all contribute to the increased prevalence of FC.^[14]^ Similarly, increased intake of whole fruits, especially fruit fiber can protect colonic gastrointestinal health.^[15]^ Additionally, another report indicated that five days of dietary changes could quickly alter the human intestinal flora.^[16]^ Therefore, the correlation between diet and constipation may be explained by changing the intestinal flora.

LVD is a set of dietary programs summarized in many years of clinical experience. The light diet means low fat, low stimulation and not prominent in cold, hot, sour, bitter, sweet, spicy, and salty; The vegetarian diet means meat, eggs and milk should be avoided and vegetables or fresh fruits should be ingested as much as possible. Several studies have proved the above mentioned food to be related with constipation.^[14]^ The diet can maintain the therapeutic effects in both subtypes of constipation after drug withdrawal, thus long-term diet intervention is realizable.^[17,18]^ To date, there is no rigorous evidence to support the effectiveness and safety of LVD, thus this needs authentication based on clinical trials in human subjects. In this protocol, we will design a randomized controlled trial (RCT) to systematically verify that point.

## 2 Methods and design

### 2.1 Study objective and hypotheses

This exploratory study aims to investigate the therapeutic effects of LVD and its supplementary effects to drugs on FC with gastrointestinal damp-heat syndrome.

### 2.2 Study design

**Figure 1:**
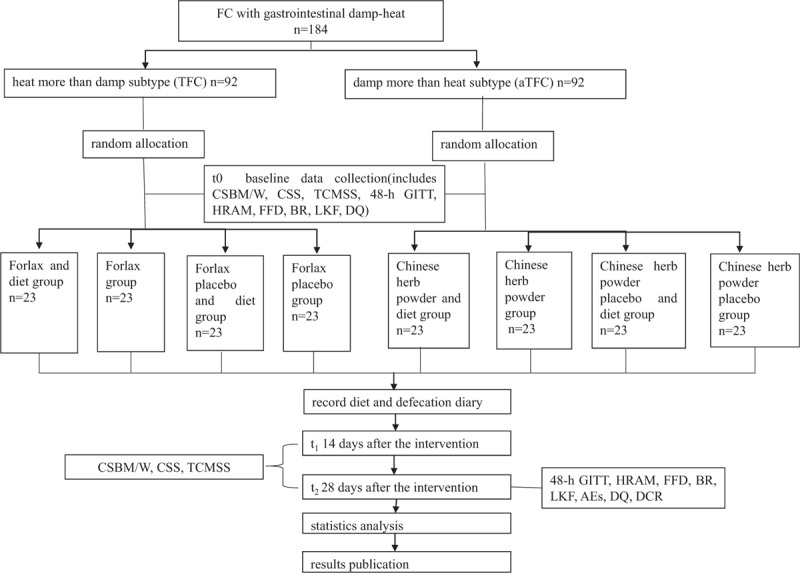
Study design. CSBM/W = complete spontaneous bowel movements per week, CSS = constipation-related symptom rating scale, TCMSS = traditional Chinese medicine syndrome scale, 48-h GITT = 48-hour gastrointestinal transit time, HRAM = high resolution anorectal manometry, FFD = fecal flora detection, BR = blood routine, LKF = liver and kidney function, AEs = adverse events, DQ = diet quality, DCR = dietary compliance rate.

**Figure 2:**
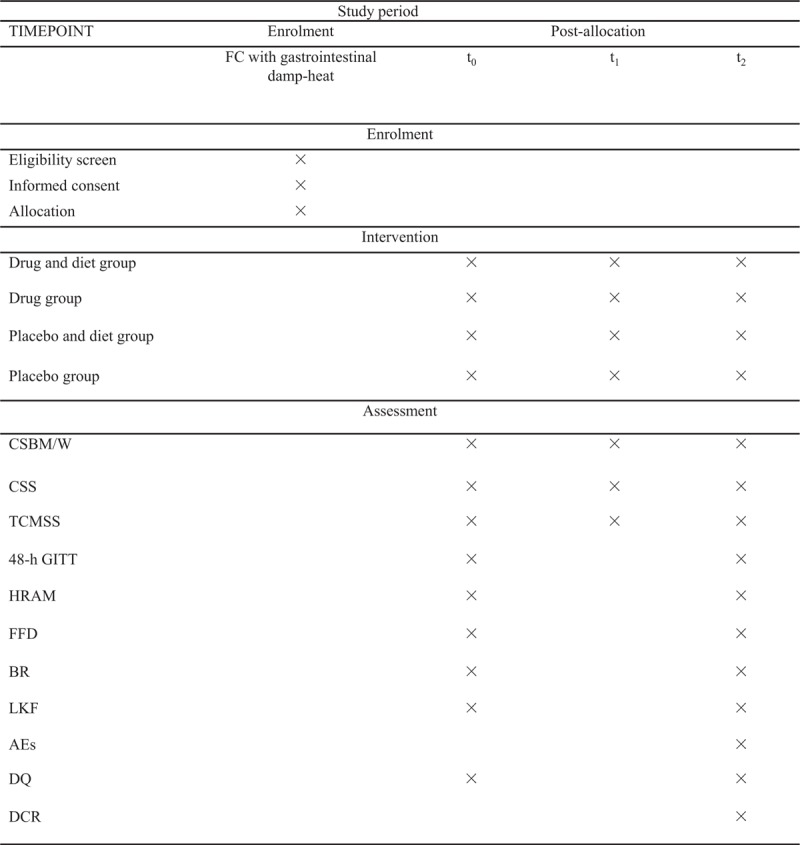
SPIRIT figure: schedule of enrolment, interventions and assessments. CSBM/W = complete spontaneous bowel movements per week, CSS = constipation-related symptom rating scale, TCMSS = traditional Chinese medicine syndrome scale, 48-hour GITT = 48-hour gastrointestinal transit time, HRAM = high resolution anorectal manometry, FFD = fecal flora detection, BR = blood routine, LKF = liver and kidney function, AEs = adverse events, DQ = diet quality, DCR = dietary compliance rate.

The protocol was drafted using the SPIRIT guidelines and the results will be reported following the checklist of the Consolidated Standards of Reporting Trials (CONSORT) statement (see Supplementary Table 1). All the participants will be informed about the study purpose and their signed informed consent will be obtained before joining this parallel-group study. A total of 92 participants will be recruited in each TFC or aTFC subtype following the inclusion and exclusion criteria. Then they will be allocated to drug + diet group, placebo + diet group, drug group and placebo group randomly at 1:1:1:1 ratio. The positive drugs are Macrogol 4000 Powder (Forlax, Beaufort Ipsen Industrie) and Chinese herb powder for TFC and aTFC groups respectively. Outcomes will be evaluated at baseline, the 14th day (followed-up by telephone) and 28th day by researchers blinded to the groups. Participants will be informed whether diet intervention is acted by the enrollment number and obtain the “drug” corresponding to the number, but they and the researchers will not know whether they are receiving a drug or a placebo. The whole study design is illustrated (see Fig. 1). A SPIRIT figure for the schedule of enrolment, interventions and assessments is also presented (see Fig. 2).

### 2.3 Exploratory study

LVD is original and RCT on LVD has not been investigated so far, thus it is highly innovative. Furthermore, studies on 2 subtypes of the major syndrome of FC can supplement the deficiency in traditional understanding of FC.

### 2.4 Diagnostic criteria of FC with damp-heat syndrome

Diagnosis of FC with damp-heat syndrome must satisfy both Rome IV criteria of FC and TCM criteria of gastrointestinal damp-heat syndrome (see Supplementary Table 2),^[7]^ referring to the relevant syndrome provisions of the Guiding Principles of Clinical Research on New Drugs of TCM.^[19]^

### 2.5 Inclusion criteria, exclusion criteria, and exit criteria

Participants must meet inclusion criteria (see Table 1) and be excluded from exclusion criteria (see Table 1), while exit criteria (see Table 1) are set for participants who cannot complete the study. Once a patient is included in the trial, the researchers in our team will keep in touch with him/ her and try to solve the problems during the treatment to reduce the loss of the intervention. During the trial, patients have the right to withdraw at any condition and will not affect subsequent treatment. The data will be recorded truthfully for further analysis.

**Table 1:**
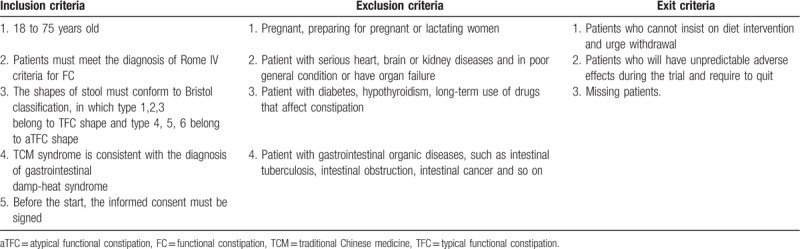
The inclusion criteria, exclusion criteria, and exit criteria for the trial.

### 2.6 Sample size

Due to the lack of relevant evidence, this is an exploratory study with small sample size. A total of 92 participants for each subtype will be recruited and will be further divided in four sub-groups (23 participants in every sub-group).

### 2.7 Recruitment

Participants will be recruited from the outpatients and inpatients of Gastroenterology Department and TCM Gastroenterology Department in China-Japan Friendship Hospital in Beijing through roll-up banners, posters, Wechat and the hospital official website. Two or more experts (at least 1 from Gastroenterology Department and 1 from TCM Gastroenterology Department) will decide the eligibility unanimously following the inclusion and exclusion criteria. It will be insured that both physicians and patients have no financial interests. All eligible recruiters will sign a written informed consent and those who accept the purpose, benefits and adverse effects of the study, will be enrolled.

### 2.8 Interventions

After the patients are enrolled, they will finish a questionnaire (baseline information) and the relevant examinations (48-h GITT, HRAM, FFD, BR, LKF). Previous studies have reported Forlax and Chinese herb as effective drugs for the treatment of TFC^[9]^ and aTFC^[20]^ respectively. So, the drug used for the treatment of TFC subtype is Forlax. The placebo will be a kind of white powder made from orange essence, saccharin sodium and dextrin (Shanghai Aipu Food Industry Corporation) according to 0.2:0.015:100 (w/w) and is similar to Forlax in shape and color. Both Forlax and placebo will be vacuum packaged as 10 g per pack using same type of aluminum foil. The drug in aTFC subtype will be Chinese herb powder dried by Chinese herb decoction (Tongrentang Chinese Medicine) from clinical prescription (see Table 2), and the placebo will be a kind of brown powder made from caramel pigment and dextrin (Shanghai Aipu Food Industry Corporation) at 0.4:10 (w/w). Both the Chinese herb powder and placebo will be vacuum packaged as 15 g per pack using same type of aluminum foil. The drugs and placebos will be taken twice a day, one pack at a time, flushed with warm boiled water, half an hour after breakfast and dinner. The contents of LVD are as following:

(1) Taboo spicy (especially chili, pepper), irritating food, wine, fried food, grilled food, dry nuts, mutton, leek, toonasinensis and so on.
(2) Avoid meat, eggs, milk, drinking coffee and strong tea.
(3) The staple food are cereals and grains, take vegetables and fruits as much as possible.
(4) Eat medicinal gruel: millet (main ingredients), coix seed, red bean that are mixed and cooked and eaten 1 to 2 times a day.
(5) More than half of the staple food is potato (such as sweet potato and potato).
(6) Diet must accord with three meals a day, regular quantitative with abstinence from drinking

**Table 2:**
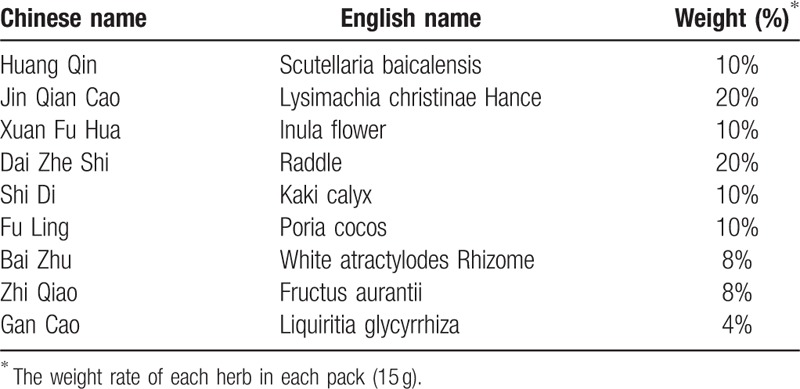
The component of Chinese herb powder.

Patients with diet intervention should follow the above items and patients without diet intervention will continue their previous eating habits. The treatment period will last for 28 days. During the treatment period, patients will be required to keep a diary about diet and defecation. The diet diary will be designed with a semi-quantitative food frequency method, and the defecation diary will gather information about sensation, stool shape and duration of each defecation. On the 14th day, patients will be followed up via telephone to find out the situation during the treatment and count the average times of CSBM per week, the average scores of constipation symptoms and TCM syndrome in the first 14 days. After 28 days of treatment, a second follow-up will be carried out to understand situation of constipation (the same to the first follow-up) and to ask the patients to finish the examinations (48-hour GITT, HRAM, BR, LKF). Fecal flora of fresh stool (<30 minutes) from patients will also be reviewed and the diaries will be taken back. During the intervention, the patients will keep in touch with the researchers for queries about the treatment if any to control the exit rate and any other drugs that can relieve constipation are forbidden.

### 2.9 Randomization, allocation concealment and partly double-blind

Two groups, each one of 92 independent random numbers, will be generated using Excel 2013 software (Microsoft Corporation, Redmond, Washington) by the third party that does not participate in the study, and will be divided by 4. The remainder will be 1 for group A, 2 for group B, 3 for group C and 0 for group D. A, B, C, D are prescribed as diet + drug, diet + placebo, drug and placebo group, respectively. The number of the drug will be reserved and will be encapsulated and stored in opaque envelopes, preserved by the third party. When a patient is enrolled, an envelope with a sequence number on its surface will be passed to the researchers. The researchers and patients will know whether diet is interfered and the drug number only, but not the corresponding drug or placebo group. When the trial is finished, the third party will uncover the blindness. It is difficult to conduct blinding since the diet intervention is a set of dietary principles, rather than specific food, so only drugs are double-blind to reduce the information bias.

### 2.10 Emergency unblinding

In case of any serious adverse events (SAEs) or emergency, the researcher will report to the supervisor and the main researcher to decide whether to open the emergency letter. Once opened, the case will be treated as exfoliated, excluded from the efficacy analysis, but will remain included in the adverse reaction analysis. Details of the unblinding cause, date, treatment situation and results will be reported in the case report form (CRF) and will be signed by the administrator.

### 2.11 Quality control

Participants’ DQ will be assessed at the baseline and after the intervention. The foods involved are in LVD content.

### 2.12 Data collection

#### 2.12.1 Baseline information

Baseline information will include demographic data and clinical data from the participants after allocation. Demographic data includes gender, age, height, weight, nationality, occupation, and education level. Clinical data includes past medical history, drug allergy history, the history of medications used to treat or induce constipation, dietary habits in the past year (see Supplementary Table 3),^[21]^ the primary outcome and secondary outcomes.

#### 2.12.2 Primary outcome

The number of CSBM per week is the primary outcome. It is effective when the number before the intervention subtracted from the number after the intervention is more or equal to 1.

#### 2.12.3 Secondary outcomes

*CSS:* The scale for constipation-related symptoms is used for investigating the degree and frequency of symptoms mentioned in Roman IV diagnostic criteria for FC. The degree of discomfort is assessed by visual analog scale (VAS), in which 0 represents no discomfort and 10 represents very strong discomfort. The final scores are obtained with the multiplication of the 2 and then divided by the average number of defecations per week (see Supplementary Table 4). Percent improvement will be calculated as following:
 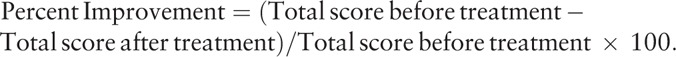


Recovery means symptoms disappeared; Marked improvement means percent improvement is more than or equal to 80%; Progress means percent improvement is more than or equal to 50% but less than 80%; Ineffective means percent improvement is less than 50%.

*TCMSS:* The TCMSS is established by collecting information about effects of relevant syndromes of TCM constipation, which are assigned 0, 1, 2, and 3 points representing normal, mild, moderate, and severe degrees, respectively. Normal refers to the absence of this syndrome, mild refers to a slight impact on life, moderate means a significant impact on life and severe refers to the serious impact on life (see Supplementary Table 5). Nimodipine method is used for evaluating the effects of relevant syndromes of TCM constipation.
 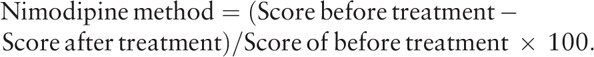


Symptoms, signs disappeared or basically disappeared and syndrome integral reduced more than or equal to 95% is considered clinical cure; Significantly effective means that symptoms, signs significantly have improved and syndrome integral has reduced more than or equal to 70%; Effective means that symptoms, signs have improved and syndrome integral have reduced more than or equal to 30%; Ineffective means that symptoms, signs have no improved or have even aggravated and syndrome score has reduced less than 30%.

*48-h GITT:* 48-h GITT is a simple method for evaluating the function of gastrointestinal motility. The barium residue rate is the ratio of the number of barium remaining in the body 48 hours after swallowing 20 barium bars with a standard meal. Higher residue rate indicates the worse gastrointestinal transport function. The parameters include barium excretion rate after 48-hour, residue rate above rectosigmoid colon and residue rate within rectosigmoid colon. The barium excretion rate in normal people is more than or equal to 90%.

*HRAM:* HRAM is used to estimate the function of anal sphincter, rectal sensation and anorectal reflex for diagnosing the defecation disorder constipation. The parameters include mean pressure of resting anal sphincter, maximum contraction pressure of resting anal sphincter, anal relaxation rate during analog defecation, first sensory value, defecation sensory value and maximum tolerance.

*FFD:* Fresh stools (<30 minutes, no drugs influencing on intestinal flora were taken in the past 2 weeks) from the participants are stored in a refrigerator at –80°C for flora detection. 16S rDNA sequencing is used to detect the distribution of fecal flora at the genus level.

#### 2.12.4 Safety outcomes

Safety parameters include AEs and laboratory examinations (BR, LKF).

#### 2.12.5 DQ and compliance measure

The number of qualified meals, total meals and the intake frequency of food involved will be calculated after getting the diary back. Diet high quality means a significant difference before and after intervention in diet groups, while there is no difference in non-diet groups. DCR means the ratio of qualified meals to total meals multiplied by 100. High compliance is achieved when DCR is more than or equal to 80%, while low DCR is less than 80%.

### 2.13 AEs

AEs mean negative effects caused by the treatment. During the study, any abnormal reactions from participants will be reported to the researchers. The occurrence time, side effects, degree, duration and mitigation will be recorded in the CRF.

### 2.14 Data monitoring and confidentiality

Researchers will be trained in a unified way before the study to ensure it is performed correctly. Data will be locked in the airtight cabinet and will be entered and checked by double entry into the database established by data administrator using Excel 2013 edition (Microsoft Corporation, Redmond, Washington) for management after completion of the study. An independent statistician will analyze the data while the procedure will be monitored by a third individual. The data of the patients involved in the trial will be confidential and will be used only by scientific research institutes. The publication or reporting of any research findings will not reveal the individual identity.

### 2.15 Planned statistical analysis

Continuous variables will be described as the mean ± standard deviation (SD), median or interquartile range (IQR). The number of cases, frequency, the rate and the constituent ratio will be described by categorical variables. Variance analysis will be used for continuous variables in the baseline data having normal distribution and same variance. Wilcoxon rank sum test or Kruskal–Wallis *H* test will be used for the comparison of differences between groups that are not normally distributed or have uneven variance. Categorical variables will be tested by chi–square test or Fisher exact test. Wilcoxon rank sum test or Kruskal–Wallis *H* test will be used for the number of CSBM per week in primary outcome. Chi–square test or Fisher exact test will be used for percent improvement of each symptom scale score in secondary outcome indicators. The result of 48-h GITT will be analyzed using chi-square or Fisher test. Wilcoxon rank sum test or Kruskal–Wallis *H* test will be used for measurement data in HRAM, while chi-square test or Fisher exact test will be used for counting data. Safety, dietary quality, compliance indicators will be tested by chi-square test or Fisher exact test. Wilcoxon rank sum test or Kruskal–Wallis *H* test will be used to compare the diversity, the relative abundance of bacteria in phylum and genera level between 2 or more groups.^[22]^ Complying with the intent-to-treat (ITT) and per-protocol (PP) principle, all data of the patients randomized and at least 1 assessment (include baseline assessment) will be analyzed. For dropouts and terminations, their missing data will be obtained by last-observation-carried-forward (LOCF) method. All statistical analyses will be performed by SPSS20 software (IBM, Chicago, Illinois), and *P* value <.05 will be considered statistically significant.

### 2.16 Ethics and dissemination

This study has been approved by China-Japan Friendship Hospital clinical research ethics committee (No. 2017–46–1). All participants will need to sign the informed consent before the starting the study. During the intervention, patients have the right to withdraw at any condition and will not affect subsequent treatment (personal treatments from the expert team). We must submit a written application to the ethics committee if the protocol needs to be modified. The committee members will decide whether to modify it. The results of the study will be sent to researchers, participants and disseminated to the public through academic conferences and journals. This is an open access article, which permits unrestricted use when the original work is correctly cited.

## 3 Discussion

FC is a common disease of the digestive system and is mainly characterized by persistent or recurrent difficulty in defecation. Other digestive symptoms such as loss of appetite, abdominal distention, hemorrhoids, and cardiovascular and cerebrovascular diseases or even sudden death are also associated with FC.^[23]^ TCM theory explains well FC not only with dry or lumpy stool (TFC) but also with sticky even unshaped stool (aTFC). Previous studies have reported Forlax and Chinese herb as effective drugs for the treatment of TFC^[9]^ and aTFC respectively.^[20]^ Unfortunately, these drugs therapies can only improve constipation provisionally, and difficult defection recurs after drug withdrawal. Diet control may provide a solution to the current situation. Many studies have shown that the supplementation of dietary fiber could improve constipation.^[24,25]^ The daily diet could provide enough dietary fiber without additional intake when inappropriate dietary structure is changed. LVD is based on vegetarian diet, eliminating spicy and stimulating food.^[26]^ Meanwhile, low-fat, sodium-restricted, protein-restricted diets are also recommended.^[27,28]^ Maintenance in improvement for FC has been observed in clinical practice for patients who have insisted on LVD for a period of time without any medication.^[17]^ However, these are only the results of clinical observations and need to be scientifically authenticated based clinical trials. This is the first RCT to illuminate the effectiveness and safety of LVD on FC with gastrointestinal damp-heat. Results of this exploratory study can provide reliable evidence of dietary efficacy, and will also clarify the supplementary effect of diet to drug. But there still remain some limitations:

(1) It is difficult to perform blind for diet intervention because diet is a non-pharmacologic factor.^[29]^
(2) This trial can explore the effect and supplementary effect of diet, but the interaction between diet and drug is not yet investigated.
(3) The sample size is kept small due to the exploratory nature of the current study.
(4) In previous studies of our research group, there is no adequate proof of the effectiveness of Chinese medicine in TFC subtype, while Forlax is a well-established positive drug.

Thus, a multicenter RCT with large sample size should be designed to verify the effectiveness and safety of LVD on FC, and some basic studies need to be performed for investigating it on mechanistic level in the future.

## Acknowledgments

The authors thank the physicians and other workers of the Department of Gastroenterology and TCM Gastroenterology in China-Japan Friendship Hospital to assist the trial to carry out. Yu Liu and Xudong Gao are co-first authors, they contributed equally. They also thank Dong Li from the Pharmaceutical Department in China-Japan Friendship Hospital to assist the drug and placebo packed. They thank Ruihua Sun from the Clinical Institute of China-Japan Friendship Hospital for giving guidance on statistics.

## Author contributions

**Conceptualization:** Yu Liu, Shukun Yao

**Data curation:** Yu Liu, Yuehua Ding, Yuanchen Zhou, Xinyuan Liu

**Formal analysis:** Yu Liu, Xudong Gao, Yuehua Ding, Yuanchen Zhou,

**Funding acquisition:** Shukun Yao, Xudong Gao

**Investigation:** Yuehua Ding, Yuanchen Zhou, Xinyuan Liu, Huijing Wang, Qianqian Wang

**Methodology:** Yu Liu, Bingzhi Ma, Shukun Yao

**Project administration:** Shukun Yao

**Software:** Xudong Gao, Huijing Wang, Qianqian Wang

**Supervision:** Shukun Yao

**Writing – original draft:** Yu Liu, Xudong Gao

**Writing – review & editing:** Shukun Yao

## Supplementary Material

Supplemental Digital Content

## Supplementary Material

Supplemental Digital Content

## Supplementary Material

Supplemental Digital Content

## Supplementary Material

Supplemental Digital Content

## Supplementary Material

Supplemental Digital Content
