# Supplementary material for: Effectiveness and safety of light vegetarian diet on functional constipation with gastrointestinal damp-heat pattern: An exploratory study protocol for randomized controlled trial
Source: Medicine (Baltimore). 2019 Dec 16;98(50):e18325. doi: 10.1097/MD.0000000000018325 (PMC6922355; doi:10.1097/MD.0000000000018325)
Supplement: Supplemental Digital Content [file medi-98-e18325-s002.docx]

Table 2

Diagnostic criteria of TCM: Referring to the relevant provisions of the Guiding Principles for Clinical New Drugs of Traditional Chinese Medicine

| **1. Gastrointestinal damp-heat syndrome (damp > heat)：** |
| --- |
| - 1. Primary symptoms:   1.1.1Sticky stool 1.1.2.Abdominal fullness and distention 1.1.3.Poor appetite 1.1.4.Thirsty and drinking less 1.1.5.Red tongue and yellow and greasy fur |
| 1.2 Secondary symptoms:  1.2.1 Heavy body trapped 1.2.2.Hiding fever or fever after sweating 1.2.3.Abdominal distension 1.2.4. Nausea and vomiting 1.2.5.Body and eyes yellow and bright 1.2.6.Slippery and rapid pulse. |
| Above them, there are 3 primary symptoms (1.1.1, 1.1.5 are essential) or 2 primary symptoms (1.1.1, 1.1.5 are essential) and 2 secondary symptoms, which can be diagnosed. |
| **2. Gastrointestinal damp-heat syndrome (heat > damp):** |
| 2.1Primary symptoms:  2.1.1. Hard or dry stool 2.1.2.Red tongue with yellow and slightly greasy coating |
| 2.2 Secondary symptoms:  2.2.1. Abdominal distention or pain 2.2.2.Thirst and ozostomia 2.2.3. Upset and insomnia 2.2.4. Short and yellow urine 2.2.5. Rapid and slippery pulse |
| Above them, when the primary symptoms are necessary and two or more secondary symptoms, the diagnosis can be made. |

Effectiveness and safety of light vegetarian diet on functional constipation with gastrointestinal damp-heat pattern: an exploratory study protocol for randomized controlled trial, Yu Liu
